# COVID-19 Diagnosis from CT Images with Convolutional Neural Network Optimized by Marine Predator Optimization Algorithm

**DOI:** 10.1155/2021/5122962

**Published:** 2021-10-12

**Authors:** Huaping Jia, Junlong Zhao, Ali Arshaghi

**Affiliations:** ^1^College of Computer, Weinan Normal University, Weinan, Shaanxi, China; ^2^Rehabilitation Medicine Department, Weinan Central Hospital, Shaanxi, China; ^3^Department of Electrical Engineering, Central Tehran Branch, Islamic Azad University, Tehran, Iran

## Abstract

In recent years, almost every country in the world has struggled against the spread of Coronavirus Disease 2019. If governments and public health systems do not take action against the spread of the disease, it will have a severe impact on human life. A noteworthy technique to stop this pandemic is diagnosing COVID-19 infected patients and isolating them instantly. The present study proposes a method for the diagnosis of COVID-19 from CT images. The method is a hybrid method based on convolutional neural network which is optimized by a newly introduced metaheuristic, called marine predator optimization algorithm. This optimization method is performed to improve the system accuracy. The method is then implemented on the chest CT scans with the COVID-19-related findings (MosMedData) dataset, and the results are compared with three other methods from the literature to indicate the method's performance. The final results indicate that the proposed method with 98.11% accuracy, 98.13% precision, 98.66% sensitivity, and 97.26% *F*1 score has the highest performance in all indicators than the compared methods which shows its higher accuracy and reliability.

## 1. Introduction

During authoring this paper on May 15, 2021, 162,974,265 COVID-19 cases and 3,378,495 deaths are reported by the “Worldometers” organization. This disease was officially named by the World Health Organization as coronavirus disease 2019 (COVID-19) on the 11th of February 2020. The outbreak, originally associated with a city in China, has now become a widespread pandemic, affecting more than 1.2 million people in more than 200 countries and regions around the world.

Several approaches have been introduced for diagnosing COVID-19, including nucleic acid test (NAT), chest radiographs, and CT scan of the lungs. NAT is used to identify specific nucleic acid sequences and species of an organism, mainly viruses or bacteria that cause disease in the blood, tissue, or urine. Although diagnostic kits play an important role in the diagnosis of COVID-19, chest radiographs and CT scans of the lungs are some of the most effective ways to diagnose the severity and degree of pneumonia that may have been transmitted by severe acute respiratory syndrome coronavirus 2 (SARS-CoV-2). Recently, some researches have been done on lung CT scan images for the early detection of COVID-19 based on image processing and artificial intelligence techniques.

Ahuja et al. proposed a method for the diagnosis of COVID-19 based on decomposing the CT scan images into three levels using a stationary wavelet [1]. This three-phase diagnosis system was presented to progress the accuracy diagnosis [2]. The method first used data augmentation using stationary wavelets. Then, COVID-19 was diagnosed based on the pretrained CNN model for abnormality localization in CT scan images. The method used some well-known pretrained architectures, like ResNet18, ResNet50, ResNet101, and SqueezeNet, for the diagnosis. The simulation results showed that the empirical assessment approves that the ResNet18 pretrained transfer learning-based method provides better classification accuracy.

Maghdid et al. introduced a method for diagnosing COVID-19 based on deep learning [3]. Due to the less values of the CT scan dataset for COVID-19, the study built a general dataset of CT scans and X-ray images from multiple sources to offer a simple and effective diagnosis system for COVID-19.

Then, a simple convolution neural network (CNN) and a modified pretrained AlexNet model have been performed on the datasets. The experimental results indicated that the employed models offer high accuracy for the diagnosis of COVID-19.

Minaee et al. presented a method based on analysis of radiology images for the diagnosis of COVID-19 [4]. The method had been performed on the COVID-19 chest X-ray images on the datasets from the internet. Four different structures of convolutional neural networks, including ResNet18, ResNet50, DenseNet-121, and SqueezeNet, were utilized for the diagnosis. Simulation results showed that all CNN models provide a satisfying accuracy for the diagnosis of COVID-19 disease.

Some other models based on CNN, such as the combined deep convolution networks [5] and unsupervised learning [6], are also presented for the diagnosis of COVID-19, although the method accuracy for the diagnosis of COVID-19, particularly for low-density areas, is low.

In the present study, a new method has been proposed for COVID-19 area segmentation based on a CNN architecture using VGG-16 encoder for semantic and U-Net segmentation methods. The presented methodology does not need more training data owning to the advantages of U-Net, which provides a model to be used on systems with low-strength GPUs. Therefore, the main contribution of this study can be highlighted as follows:

- Proposing a new optimal method for the diagnosis of COVID-19 from CT images
- Using a hybrid technique based on convolutional neural network (CNN) and metaheuristic techniques
- Optimizing the CNN based on a newly introduced metaheuristic, called marine predator optimization algorithm

## 2. Convolutional Neural Network

Since the advent of deep learning, the convolutional neural network (CNN or ConvNet) has been the flagship of ideas in deep learning [7]. The CNN was introduced in 1990, inspired by experiments performed by Hubel and Wiesel on the visual cortex. The CNN is a modified version of an artificial neural network that can be employed for various mathematical learning methods such as backpropagation, gradient descent, and regularization [8, 9]. Due to the CNN's special structure and filter-like state, it is processed in the signal area. This network includes three main concepts of layers with a convolutional layer, pooling layer, and fully connected layer.

In a CNN, different layers perform different tasks with two steps for training: the feed-forward stage and the backpropagation stage. In the first stage, the input image is fed to the network and this action is nothing but multiplying the point between the input and the parameters of each neuron and finally applying a convolution operation in each layer. The network output is then calculated. Here, to adjust the network parameters or in other words the network training, the output result is used to calculate the amount of network error. To do this, the output of the network is compared with the correct solution based on the loss function to calculate the error rate. In the next step, based on the calculated error rate, the backpropagation step begins. In this step, the gradient of each parameter is calculated according to the chain rule and all parameters change according to the effect they have on the error created in the network. After updating the parameters, the next feed-forward step begins. After repeating a proper number of these steps, the network training ends.

The learning process in the CNN is to obtain kernel matrices to generate better features of the problem (here, COVID-19 diagnosis). The backpropagation (BP) technique has been considered for learning and for minimizing the error value of the network. The study uses a sliding window for convolution.

The activation function is a rectified linear unit (ReLU) such that *f*(*x*) = max(*x*, 0) [10]. The method of scale reduction in this study is max pooling. BP defines a gradient descent technique to minimize the error of the neural network by minimizing the cross-entropy [11] which can be mathematically formulated as follows:

(1) $$\begin{array}{c} L=\overset{N}{\underset{j=1}{\sum }}\overset{M}{\underset{i=1}{\sum }}-d_{j}^{\left(i\right)}\log z_{j}^{\left(i\right)}, \end{array}$$

where *N* signifies the number of samples, $d_{j}=\left(0,\cdots ,0,\underset{k}{\underset{⏟}{1,\cdots ,1}},0,\cdots ,0\right)$ describes the desired output vector, and *z*_*j*_ defines the achieved output vector of the *m*^th^ class that is achieved as follows:

(2) $$\begin{array}{c} z_{j}^{\left(i\right)}=\frac{e^{f_{j}}}{\sum_{i=1}^{M}e^{f_{i}}}. \end{array}$$

**Figure 1:**
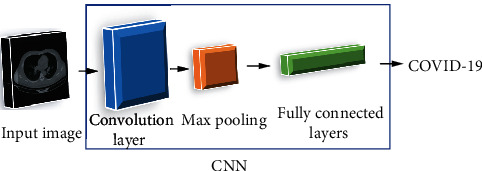
A block diagram of a simple CNN for COVID-19 diagnosis.

The function has been extended based on a weight penalty by considering *η* term as follows:

(3) $$\begin{array}{c} L=\overset{N}{\underset{j=1}{\sum }}\overset{M}{\underset{i=1}{\sum }}-d_{j}^{\left(i\right)}\log z_{j}^{\left(i\right)}+\frac{1}{2}\eta \underset{K}{\sum }\underset{L}{\sum }\omega_{k,l}^{2}, \end{array}$$

where *L* signifies the total number of layers, *K* is the layer *l* connections, and *ω*_*k*_ describes the weight for connection.  Figure 1 shows a block diagram of a simple CNN for COVID-19 diagnosis.

Several research works have been proposed to optimize the arrangement of the convolutional neural network. Particularly, the application of optimization algorithms in CNNs indicated satisfying achievements [12]. The present study uses a new optimal technique to provide an optimized CNN. All input images have been resized to 28-by-28 pixel images to improve the speed of diagnosis. For the COVID-19 diagnosis problem, the CNN arrangement should be explained by considering some terminology depending on the suggested CNN arrangement:

- The input layer of the image in which the input images of the network are normalized by the process
- 2D convolutional layer which implements sliding convolutional filter to convolve with the input image by striding the filter along with the input image horizontally and vertically and evaluates the dot product of the weights and the input image. A bias term is then also added
- Batch normalization layer which is used for normalizing the input channels of the input image crosswise minibatch
- ReLU layer which makes a threshold operation to discard negative values of the image
- 2D max pooling layer which makes downsampling by dividing the input image into rectangular pooling regions by calculating the maximum of the regions
- 2D max unpooling layer which unpools the output of the max pooling layer
- Softmax layer which performs the softmax function on the input image

**Figure 2:**
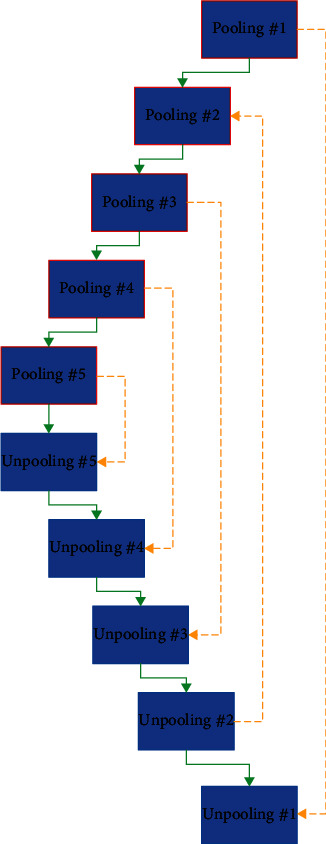
The main architecture of the proposed CNN.

The suggested CNN for COVID-19 diagnosis contains five max pooling and unpooling layers. The main architecture of the CNN is shown in Figure 2.

As can be observed from Figure 2, the layer order for Pooling #1 defines an image input layer, 2D convolution layer, batch normalization layer, ReLU layer, and 2D max pooling layer.

For block Pooling #2, the order is 2D convolution layer, batch normalization layer, ReLU layer, 2D convolution layer, batch normalization layer, ReLU layer, and 2D max pooling layer.

For block Pooling #3, Pooling #4, and Pooling #5, the order is 2D convolution layer, batch normalization layer, ReLU layer, 2D convolution layer, batch normalization layer, ReLU layer, 2D convolution layer, batch normalization layer, ReLU layer, and max pooling 2D layer.

For block Unpooling #5, Pooling #4, and Unpooling #3, the order is 2D max unpooling layer, 2D convolution layer, batch normalization layer, ReLU layer, 2D convolution layer, batch normalization layer, ReLU layer, 2D convolution layer, batch normalization layer, and ReLU layer.

For block Unpooling #2, there are 2D max unpooling layer, 2D convolution layer, batch normalization layer, ReLU layer, 2D convolution layer, batch normalization layer, and ReLU layer. For block Unpooling #1, 2D max unpooling layer, 2D convolution layer, batch normalization layer, ReLU layer, softmax layer, and classification output layer (pixel classification layer) have been used. The presented CNN architecture employed a VGG-16 encoder with U-Net construction.

## 3. Marine Predator Optimization Algorithm

There are two types of optimization algorithms: exact algorithms and approximate algorithms [13]. Exact algorithms as the first priority present the exact optimal solutions for optimization problems; thus, they are not well organized for hard optimization problems, such that their execution time improves exponentially based on the problem dimensions [14]. By using approximate algorithms, suitable solutions with a short period can be achieved for optimization problems, even for NP-hard optimization problems that cannot be solved by the exact methods [15]. Metaheuristic algorithms are the best candidates of approximate algorithms [16]. Metaheuristic algorithms define a kind of random algorithm that is employed to provide the optimal solution [17, 18]. Numerous metaheuristic algorithms have been presented in the last decade, e.g., World Cup Optimization (WCO) algorithm [19], Arithmetic Optimization Algorithm (AOA) [20], Ant Lion Optimizer (ALO) algorithm [21], and equilibrium optimizer [22].

Marine predator algorithm (MPA) [23] is another new metaheuristic algorithm that is introduced by Faramarzi et al. The MPA is a new metaheuristic algorithm inspired by marine predators that are used for solving optimization problems. The marine predator algorithm starts with random numbers which are spread uniformly in the search space. This is mathematically modeled as follows:

(4) $$\begin{array}{c} X_{0}=X_{\min}+\mathrm{rand}\times \left(X_{\max}-X_{\min}\right), \end{array}$$

where rand describes a uniformly distributed random number in the range [0, 1] and *X*_min_ and *X*_max_ represent the minimum and maximum boundaries.

The best predators have more intelligence for hunting based on the “survival of the fittest theory” [24]. Accordingly, the best predator is defined as “Elite,” which is appropriate for generating a matrix. The prey search has been defined based on matrix arrays using the prey information location. This is defined by the following matrix:

(5) $$\begin{array}{c} E={\left[\begin{array}{ccc} X_{1,1}^{I} & \cdots & X_{1,d}^{I}\\ \vdots & \ddots & \vdots \\ X_{n,1}^{I} & \cdots & X_{n,d}^{I} \end{array}\right]}_{n\times d}, \end{array}$$

where *d* signifies the dimensions' number, *X*^*I*^ represents the best predator vector with *n* simulation to generate the Elite matrix (*E*), and *n* is a variable to describe the number of candidates.

Both prey and predator are considered as candidates. This is because when the prey is looking for food, the predator is looking for the prey. At the end of each iteration, the best predator is updated as the new Elite. Furthermore, another matrix with a similar dimension of the Elite is generated as prey, where the position of the predator has been updated by this matrix:

(6) $$\begin{array}{c} P={\left[\begin{array}{ccc} X_{1,1} & \cdots & X_{1,d}\\ \vdots & \ddots & \vdots \\ X_{n,1} & \cdots & X_{n,d} \end{array}\right]}_{n\times d}, \end{array}$$

where *X*_*i*,*j*_ describes the *j*^th^ dimension for the *i*^th^ prey. Particularly, the optimization method is associated with these matrixes.

The MPA contains three main units around different speed ratios that are defined as follows:

- The prey moves faster (with a higher speed ratio) than the predator
- The predator moves faster (with a lower speed ratio) than the prey
- Both prey and predator move with the same velocity (with equal speed ratio)

Some phases have been clarified by nature principles of prey and predator movement with nature. This description is defined by the following:

- This step includes the exploration term of the algorithm which is employed at the initial iterations. If the predator has a higher speed ratio such that *v* ≥ 10, the optimum strategy has been used for stopping moving. This is mathematically modeled by the following equation:

(7) $$\begin{array}{c} \text{While Iter}<\frac{1}{3}\text{ Ma}{\text{x}}_{\text{Iter}},\\ \overset{⟶}{\text{stepsiz}{\text{e}}_{i}}={\overset{⟶}{R}}_{B}⊗\left({\overset{⟶}{E}}_{i}-{\overset{⟶}{R}}_{B}⊗{\overset{⟶}{P}}_{i}\right),i=1,2,\cdots ,n,\\ {\overset{⟶}{P}}_{i}={\overset{⟶}{P}}_{i}+P\times \overset{⟶}{R}⊗\overset{⟶}{\text{stepsiz}{\text{e}}_{i}}, \end{array}$$

where the sign ⊗ describes the entry-wise product and ${\overset{⟶}{R}}_{B}$ represents a vector including some random values that are generated by the Brownian movement [23]. The prey movement has been modeled with the product by prey and ${\overset{⟶}{R}}_{B}$.

*P* describes a constant value (0.5) and *R* represents uniformly distributed random values between 0 and 1, and Iter and Max_Iter_ represent the present iteration and the number of iterations.

(b) This step includes the searching of the prey and the predator for the prey. This process is a middle process between the optimization processes. In this step, the exploration attempts to convey the exploitation. Indeed, both exploration and exploitation terms are included in this step. Similarly, the candidate is divided into two parts so that one is employed for exploitation and the other for exploration. In this status, whereas the predator has a Brownian movement, the prey moves in a Lévy movement

(8) $$\begin{array}{c} \text{while}\frac{1}{3}\text{Ma}{\text{x}}_{\text{Iter}}<\text{Iter}<\frac{2}{3}\text{Ma}{\text{x}}_{\text{Iter}}. \end{array}$$

Based on this policy, with the exploitation term in the candidate,

(9) $$\begin{array}{c} \overset{⟶}{\text{stepsiz}{\text{e}}_{i}}={\overset{⟶}{R}}_{L}⊗\left({\overset{⟶}{E}}_{i}-{\overset{⟶}{R}}_{L}⊗{\overset{⟶}{P}}_{i}\right),\;i=1,2,\cdots ,\frac{n}{2},\\ {\overset{⟶}{P}}_{i}={\overset{⟶}{P}}_{i}+P\times \overset{⟶}{R}⊗\overset{⟶}{\text{stepsiz}{\text{e}}_{i}}, \end{array}$$

where ${\overset{⟶}{R}}_{L}$ describes a random value that is distributed by the Lévy movement [23].

The Lévy movement of the prey has been modeled by multiplying the prey and ${\overset{⟶}{R}}_{L}$ while the prey movement has been modeled. Therefore, for the exploration term in the individual,

(10) $$\begin{array}{c} \overset{⟶}{\text{stepsiz}{\text{e}}_{i}}={\overset{⟶}{R}}_{B}⊗\left({\overset{⟶}{E}}_{i}-{\overset{⟶}{R}}_{B}⊗{\overset{⟶}{P}}_{i}\right),\;i=1,2,\cdots ,\frac{n}{2},\\ {\overset{⟶}{P}}_{i}={\overset{⟶}{E}}_{i}+P\times \text{CF}⊗\overset{⟶}{\text{stepsiz}{\text{e}}_{i}}, \end{array}$$

where CF defines a modifiable variable to cope with the predator movement that is formulated as follows:

(11) $$\begin{array}{c} \text{CF}={\left(1-\frac{\text{Itr}}{\text{Ma}{\text{x}}_{\text{Iter}}}\right)}^{\left(2\times \text{Iter}\right)/\text{Ma}{\text{x}}_{\text{Iter}}}. \end{array}$$

(c) The final step is usually allied to improve the exploitation term. Lévy has been performed as the optimum policy for the predator with *v* = 0.1 (low speed ratio). This is modeled as follows:

(12) $$\begin{array}{c} \text{while Iter}>\frac{2}{3}\text{Ma}{\text{x}}_{\text{Iter}},\\ \overset{⟶}{\text{stepsiz}{\text{e}}_{i}}={\overset{⟶}{R}}_{L}⊗\left({\overset{⟶}{R}}_{L}⊗{\overset{⟶}{E}}_{i}-{\overset{⟶}{P}}_{i}\right),\;i=1,2,\cdots ,n,\\ {\overset{⟶}{P}}_{i}={\overset{⟶}{E}}_{i}+P\times \text{CF}⊗\overset{⟶}{\text{stepsiz}{\text{e}}_{i}}. \end{array}$$

By considering the definition of the Fish Aggregating Devices (FADs), above, 80 percent of the time of the sharks was spent close to the FADs and the remaining candidates are employed for longer jumps in various dimensions perhaps for searching the position for exploitation. Therefore, considering the jumps, avoid from stuck in the local optima points. This is formulated as follows:

(13) $$\begin{array}{c} {\overset{⟶}{P}}_{i}=\left\{\begin{array}{c} {\overset{⟶}{P}}_{i}+\text{CF}\times \left({\overset{⟶}{X}}_{\min}+\overset{⟶}{R}⊗\left(X_{\max}-X_{\min}\right)\right)⊗\overset{⟶}{U}\;\;\text{if }r\leq p_{f},\\ {\overset{⟶}{P}}_{i}+\left(p_{f}\times \left(1-r\right)+r\right)\times \left({\overset{⟶}{P}}_{r_{1}}-{\overset{⟶}{P}}_{r_{2}}\right)\;\text{if }r>p_{f}, \end{array}\right. \end{array}$$

where *p*_*f*_ describes the impact of FADs and is considered 0.2 in this study, $\overset{⟶}{U}$ describes the binary vector with arrays in the range [0, 1], *r* describes a randomly distributed value between 0 and 1, *r*_1_ and *r*_2_ represent random indices of the prey matrix, and *X*_min_ and *X*_max_ represent the vector connecting the minimum and the maximum bounds of dimensions.

## 4. Optimized CNN

In the present study, we used an optimized technique to improve the efficiency of the CNN architecture and implement a good relationship between the layers for guaranteeing a suitable diagnosis system for SARS-COV-2. As we know, the original CNN uses a gradient descent algorithm for optimizing the model parameters, which includes convolution filters and the weights of fully connected layers. Due to the significance of the last layer in classification, assigning the image into a related class is significant that is accomplished by a proper connection between the weights and the previous layers. To improve the accuracy of the diagnosis system, the last weight vector training should be optimized based on the proposed marine predator optimization algorithm. The number of candidates and the iteration number for the algorithm are considered 100 and 120, respectively.

The objective function for minimizing the CNN is mathematically formulated as follows:

(14) $$\begin{array}{c} E=\frac{1}{T}\overset{N}{\underset{i=1}{\sum }}\overset{M}{\underset{j=1}{\sum }}{\left(Y_{ji}-O_{ji}\right)}^{2}, \end{array}$$

where *N* describes the number of training samples, *M* represents the number of output layers, and *Y*_*ji*_ and *O*_*ji*_ represent the desired value and the output value of the CNN.

lHere, the half-value precision function has been established for validation of the optimized diagnosis system. The algorithm then starts to optimize the CNN structure until the stopping criteria have been obtained. The designed system is then validated and verified on a dataset based on the Mean Square Error (MSE). Then, the MSE has been minimized by optimal selection of the weights and biases, i.e.,

(15) $$\begin{array}{c} W=\left(w_{1},w_{2},\cdots ,w_{p}\right),\\ b_{n}=\left(b_{1n},b_{2n},\cdots ,b_{Ln}\right),\\ l=1,2,\cdots ,L,\\ n=1,2,\cdots ,A,\\ A=\left(a_{1},a_{2},\cdots ,a_{A}\right),\\ w_{n}=\left(w_{1n},w_{2n},\cdots ,w_{Ln}\right), \end{array}$$

where *l* describes the layer index, *A* defines the total number of candidates, *w*_in_ represents the value of the weights in the *i*^th^ layer, *L* signifies the total number of layers, and *n* defines the number of the candidates.

## 5. Dataset Description

**Figure 3:**
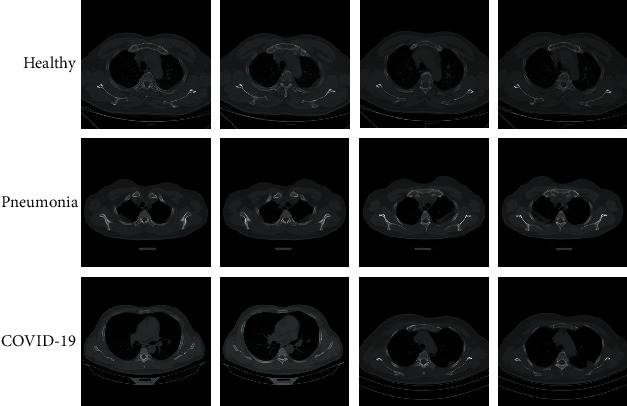
Some examples of the CT scan images collected from the MosMedData dataset [25].

The method of authentication has been presented by a standard test case of SARS-CoV-2 dataset. Numerous datasets are proposed for the diagnosis of SARS-CoV-2. The presented study uses chest CT scans with SARS-CoV-2-related findings (MosMedData) for the analysis [25]. The dataset has been collected by the Research and Practical Clinical Center for Diagnostics and Telemedicine Technologies of the Moscow Health Care Department (MosMed). 1110 patients are analyzed based on NIfTI format.  Figure 3 shows some examples of the CT scan images collected from the dataset.

After data acquisition from the dataset, to improve the quality of the raw data for statistical analysis and for increasing the accuracy of the system, some preprocessing has been done on the raw data. The first preprocessing step is data conversion. This process is a mathematical method employed for modifying variables that do not follow the statistical assumptions of linearity, normality, and uniform scattering or have patterns with uncommon outliers.

Here, data normalization has been employed. This process normalizes data/variables and puts data in the same domain when they are not. In this study, the Min–Max method has been used for the normalization. Based on the Min–Max method, unifying data scale, the data changing edges will be distributed between 0 and 1. Considering attribute *X*, so that it has a mapping from the dataset in the range [*X*_min_, *X*_max_], the Min–Max normalization ($\bar{X}$) is mathematically given as follows:

(16) $$\begin{array}{c} \bar{X}=\frac{X-X_{\min}}{X_{\max}-X_{\min}}. \end{array}$$

## 6. Simulation Results

The present study implements the training process and the proposed COVID-19 diagnosis system on MATLAB 2019b. The system configuration for the computation is Windows 10 Enterprise with Intel® Core™ i7-4720HQ, 1.60 GHz, 16 GB RAM with Intel HD GPU 4600. The main idea is to introduce a new system for the diagnosis of COVID-19. The system is assessed by four measurement indicators that contain precision, accuracy, sensitivity, and *F*1 score.

### 6.1. Accuracy

The accuracy is a measurement indicator for achieving the rate of similarity of the image with the real value. This is established by the proportion of correct identification values to the total number of identifications. This indicator is mathematically obtained as follows:

(17) $$\begin{array}{c} \text{Accuracy}=\frac{\sum_{i=1}^{l}\left(\text{T}{\text{P}}_{i}+\text{T}{\text{N}}_{i}\right)}{\sum_{i=1}^{l}\left(\text{T}{\text{P}}_{i}+\text{T}{\text{N}}_{i}+\text{F}{\text{P}}_{i}+\text{F}{\text{N}}_{i}\right)}, \end{array}$$

where TN and FN define the true negative and false negative, respectively, and TP and FP describe the true positive and false positive, respectively.

### 6.2. Precision

Precision describes the way of similarity of the measured values to each other. This indicator is established based on the proportion of positive identification values to the total number of identifications. This is mathematically defined by the following equation:

(18) $$\begin{array}{c} \text{Precision}=\frac{\sum_{i=1}^{l}\left(\text{T}{\text{P}}_{i}+{\text{FP}}_{i}\right)}{\sum_{i=1}^{l}\left(\text{T}{\text{P}}_{i}+\text{T}{\text{N}}_{i}+\text{F}{\text{P}}_{i}+\text{F}{\text{N}}_{i}\right)}. \end{array}$$

### 6.3. Sensitivity

This indicator shows the extent of positives that are accurately detected. The sensitivity is established by the proportion of true-positive recognition values to the true-positive and false-negative number of recognition. This is mathematically modeled as follows:

(19) $$\begin{array}{c} \text{Sensitivity}=\frac{\sum_{i=1}^{l}\text{T}{\text{P}}_{i}}{\sum_{i=1}^{l}\left(\text{T}{\text{P}}_{i}+\text{F}{\text{N}}_{i}\right)}. \end{array}$$

### 6.4. *F*1 Score

This score defines the exactness of the degree of a test set. This measure is achieved by the sensitivity and precision of the test. The most notable value of an *F* score is 1, which indicates idealized exactness and review, and the least conceivable value is 0, with the chance that either the precision or sensitivity is 0. The *F*1 score is moreover recognized as the Dice similarity coefficient (DSC) and is mathematically formulated as follows:

(20) $$\begin{array}{c} F1_{\text{score}}=\frac{2\times \text{Precision}\times \text{Recall}}{\text{Precision}+\text{Recall}}. \end{array}$$

The analysis results of the defined indicators are reported in Tables 1–4. This technique is compared with three state-of-the-art techniques including Horry et al.'s [26], Li et al.'s [27], and Ahuja et al.'s [1] for better clarification.

Accuracy is 98.11%, precision is 98.13%, sensitivity is 98.66%, and *F*1 score is 97.26%.

**Figure 4:**
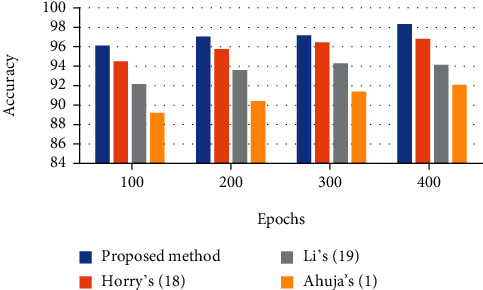
The accuracy bar plot for the assessed algorithms.

**Figure 5:**
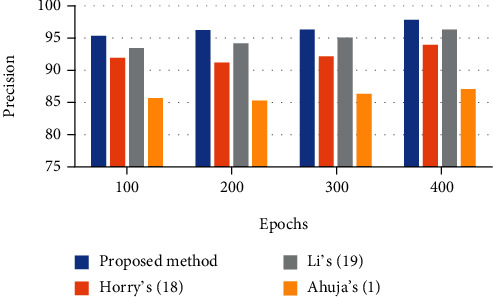
The precision bar plot for the assessed algorithms.

**Figure 6:**
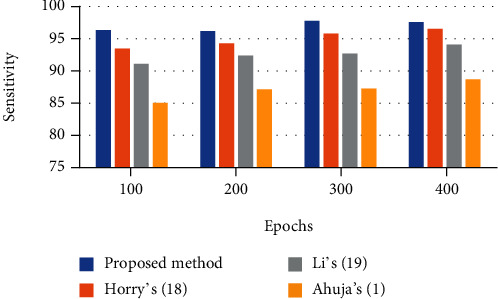
The sensitivity bar plot for the assessed algorithms.

**Figure 7:**
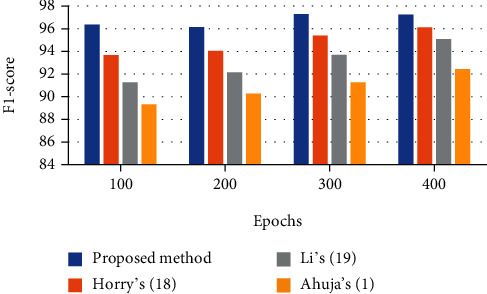
The *F*1 score bar plot for the assessed algorithms.

To provide better observation of the system effectiveness, a bar plot of the results is shown in Figures 4–7. It can be observed from Figures 4–6 that there is 98.32% accuracy, 97.83% precision, and 98.66% sensitivity for the proposed technique after 400 epochs compared with the other investigated methods. However, Horry et al.'s, Li et al.'s, and Ahuja et al.'s are in the next ranks.

400 epochs have been implemented for the algorithm. As it is observed from the figures, the suggested method provides better sensitivity to the other comparative methods. The proposed classifier provides a 97.59% sensitivity rate, whereas Horry et al.'s, Li et al.'s, and Ahuja et al.'s have 96.53%, 94.08%, and 8.68%, respectively, for 400 epochs.  Figure 7 shows the *F*1 score bar plot for the assessed algorithms.

It is also observed that after 400 epochs, the proposed method provides the highest *F*1 score value than the other comparative methods. As can be observed, the proposed technique with a 97.26% *F*1 score value offers the highest *F* measure, and Horry et al.'s, Li et al.'s, and Ahuja et al.'s with 96.13%, 95.09%, and 92.43%, respectively, are in the next ranks.

## 7. Conclusions

The COVID-19 pandemic continues as a dangerous problem for worldwide health. One significant way to stop this pandemic is to diagnose the infected patients efficiently and execute instant isolation. The infected patients with the SARS-CoV-2 virus can be detected by CT images. In the present study, a method based on optimized convolutional neural network based on metaheuristic technique was proposed for proper diagnosis of the COVID-19 CT scan images. The method used a newly introduced metaheuristic called the marine predator optimization algorithm to improve the accuracy of the proposed CNN-based diagnosis system. The proposed method was then performed on the chest CT images with COVID-19-related findings (MosMedData) dataset. Simulation results of the proposed system were compared with three other state-of-the-art methods including Horry et al.'s, Li et al.'s, and Ahuja et al.'s to indicate the method's effectiveness. Final results indicate that the proposed method with 98.11% accuracy, 98.13% precision, 98.66% sensitivity, and 97.26% *F*1 score showed the highest performance in all indicators than the compared methods. In the future work, we will work on applying a modified version of the proposed technique on chest X-ray images to determine the capability of the proposed method for the diagnosis of COVID-19 based on X-ray images and CT scan images.

## Figures and Tables

**Table 1:** The accuracy results using different techniques.

Epochs	Proposed method	Horry et al.'s [26]	Li et al.'s [27]	Ahuja et al.'s [1]
100	96.12	94.51	92.17	89.22
200	97.05	95.76	93.61	90.43
300	97.16	96.44	94.29	91.39
400	98.32	96.81	94.14	92.08
500	98.11	96.38	95.27	92.19

**Table 2:** The precision results using different techniques.

Epochs	Proposed method	Horry et al.'s [26]	Li et al.'s [27]	Ahuja et al.'s [1]
100	95.35	91.94	93.46	85.67
200	96.25	91.22	94.17	85.29
300	96.34	92.19	95.08	86.34
400	97.83	93.97	96.33	87.11
500	98.13	94.26	97.39	88.09

**Table 3:** The sensitivity results using different techniques.

Epochs	Proposed method	Horry et al.'s [26]	Li et al.'s [27]	Ahuja et al.'s [1]
100	96.37	93.46	91.11	85.04
200	96.18	94.29	92.37	87.16
300	97.80	95.81	92.69	87.26
400	97.59	96.53	94.08	88.68
500	98.66	96.74	94.16	89.37

**Table 4:** The *F*1 score results using different techniques.

Epochs	Proposed method	Horry et al.'s [26]	Li et al.'s [27]	Ahuja et al.'s [1]
100	96.38	93.68	91.28	89.33
200	96.15	94.07	92.15	90.27
300	97.29	95.39	93.69	91.26
400	97.26	96.13	95.09	92.43
500	98.34	97.35	95.02	92.56

## Data Availability

Chest CT scans with COVID-19-related findings (MosMedData) 2020 are available from https://mosmed.ai/datasets/covid19_1110.
